# Biomechanical analysis of the door-shaped titanium plate in single-level anterior cervical discectomy and fusion

**DOI:** 10.1186/s13018-023-04474-1

**Published:** 2023-12-21

**Authors:** Senli Li, Peng Yan, Yanwei Fan, Ruibo Wang, Changjiang Zhang

**Affiliations:** Second Department of Orthopedics, The Fifth Affiliated Hospital of Zhengzhou University, Zhengzhou, 450052 China

**Keywords:** Anterior cervical discectomy and fusion, Door-shaped titanium plate, Range of motion, Neutral zone, Biomechanical study

## Abstract

**Background:**

Analyse and discuss the immediate stability of the cervical spine after anterior cervical discectomy and fusion using a door-shaped titanium plate and compare it with the traditional titanium plate, to provide biomechanical evidence for the rationality and effectiveness of the door-shaped titanium plate in clinical applications.

**Methods:**

Ten adult goat C4/5 vertebral bodies were obtained, and models were prepared using denture base resin. Biomechanical experiments were performed on the specimens before internal fixation. MTS was used to conduct non-destructive biomechanical loading tests in six directions, including flexion, extension, left–right bending, and left–right torsion, recording the range of motion (ROM) and neutral zone (NZ) of each specimen. The specimens were then randomly divided into two groups: the study group was fixed with a door-shaped titanium plate, and the control group was fixed with a traditional titanium plate. ROM and NZ in each direction were measured again. After measurements, both groups were subjected to 0.5 Hz torsion loading with a torque of 2 N m for a total of 3000 cycles, followed by measuring ROM and NZ in six directions once more.

**Results:**

Compared to before fixation, ROM and NZ in both groups significantly decreased in all six directions after fixation, with statistical significance (*P* < 0.05); after fixation, the study group showed slightly lower values for various mechanical reference parameters compared to the control group, with no statistical significance (*P* > 0.05); after 3000 torsional loads, both internal fixation groups showed increased ROM and NZ compared to after fixation but to a lower extent, and no screw or titanium plate loosening was observed. Compared to before fixation, the differences were still statistically significant (*P* < 0.05), with the study group having slightly lower ROM and NZ values in all directions compared to the control group, with no statistical significance (*P* > 0.05).

**Conclusion:**

The door-shaped titanium plate exhibits mechanical properties similar to the traditional titanium plate in all directions, and its smaller size and simpler surgical operation can be used for anterior cervical endoscopic surgery, reducing surgical trauma. It is clinically feasible and deserves further research and promotion.

## Background

Cervical spondylosis is a common degenerative disease often accompanied by symptoms such as neck pain, lower limb weakness, dizziness, and other discomforts. For patients with mild symptoms, conservative treatment is initially considered in clinical practice [1–3]. However, for those who do not respond well to conservative measures for at least 6 months, surgical treatment is recommended [4]. Anterior cervical discectomy and fusion (ACDF) is one of the classical surgical approaches for treating cervical spondylosis, exhibiting excellent efficacy in managing cervical spondylotic myelopathy (CSM) and cervical spondylotic radiculopathy (CSR) [5–8]. This surgical method was originally proposed by Smith, involving the removal of the diseased intervertebral disc and the use of autologous iliac bone for interbody fusion to restore cervical spine stability. Nevertheless, this initial method has its limitations, as a series of postoperative complications, including bone block dislocation, subsidence, and pseudoarthrosis formation, adversely affect surgical outcomes [9, 10]. The introduction of the anterior cervical plate system (ACPS) further enhances the immediate stability of the cervical spine after ACDF, reducing the duration of postoperative neck bracing and improving fusion results, with a bone fusion rate of approximately 90%. Moreover, the rate of subsidence of the fusion material is significantly reduced [11–13]. Therefore, ACPS has become a commonly used internal fixation system in clinical practice and a classic reference standard for testing the stability of newly developed internal fixation devices. However, traditional titanium plates, while offering many advantages, also have numerous shortcomings. Plates such as the Katia Anterior Cervical Internal Fixation System, AO Locking Plate, Caspar Plate, and Orion Plate are relatively thick and bulky. Consequently, they often lead to postoperative swallowing difficulties and complications such as adjacent segment degeneration (ASD), with an overall complication rate ranging from 13.2 to 19.3%. High rates of swallowing difficulties significantly impact patients’ quality of life [14–18]. Therefore, reducing the thickness of ACPS while ensuring bone fusion rate has become a focal point of research for new internal fixation devices. To address these limitations, our research team developed a door-shaped titanium plate that can be used in anterior cervical endoscopic systems. This plate is thinner, more compact, and combines the nail and plate, which has been applied in clinical practice with favourable clinical outcomes. However, there is limited biomechanical research on this device. In this study, we used goat cervical vertebrae specimens to evaluate the biomechanical performance of the door-shaped titanium plate.

## Materials and methods

**Fig. 1:**
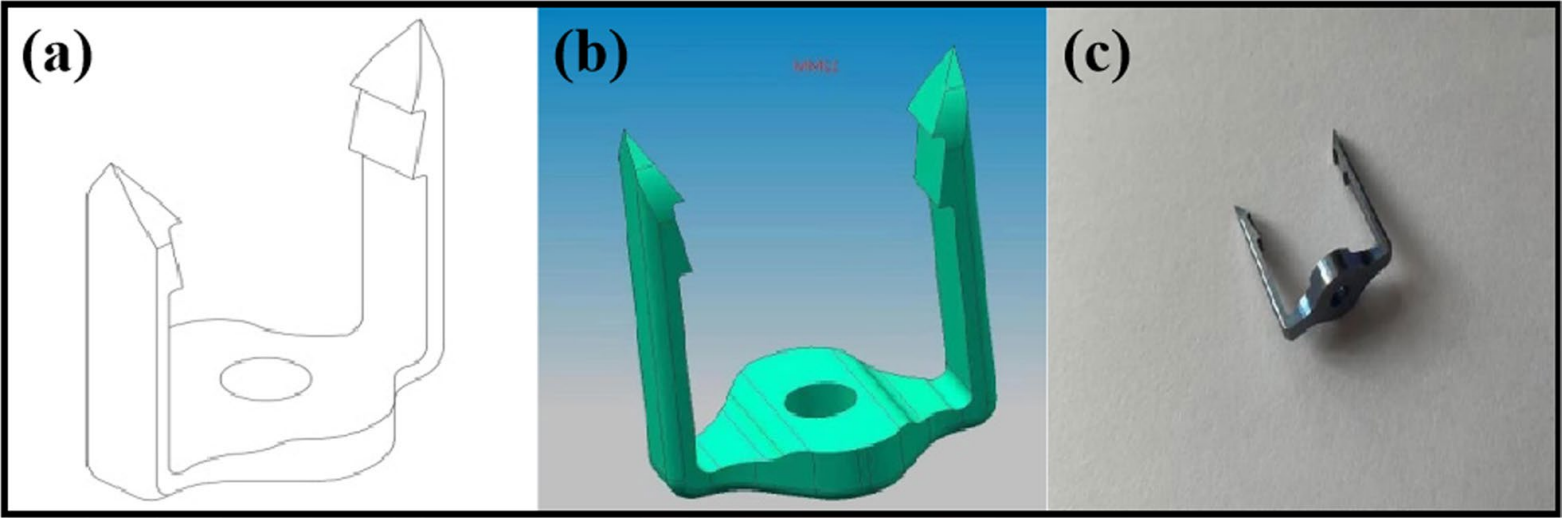
3D view and physical drawing of the door-type titanium plate

**Fig. 2:**
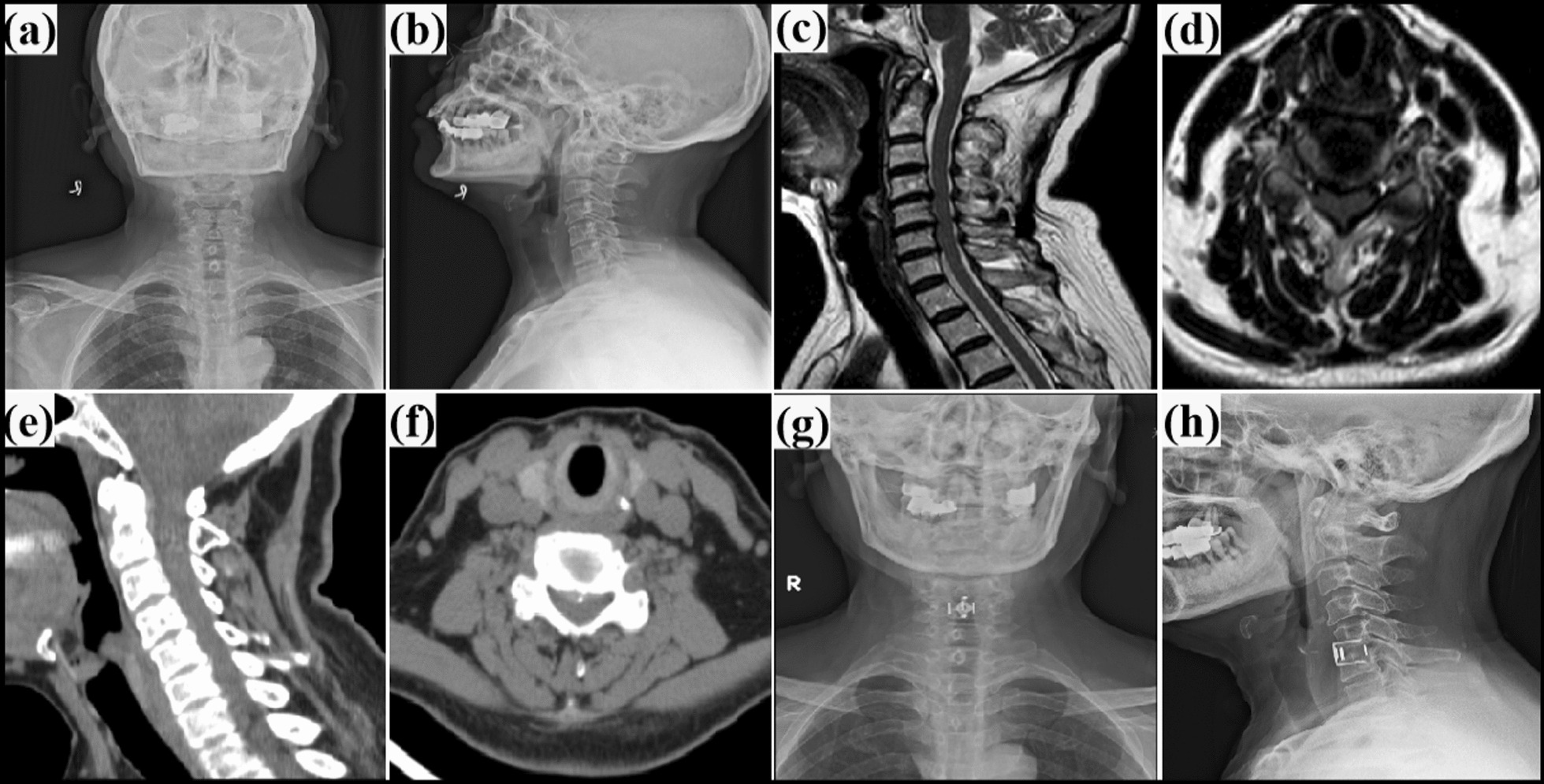
A Clinical Application Example of the Door-Shaped Titanium Plate: **a**, **b** Preoperative cervical X-ray images in anteroposterior and lateral views; **c**, **d** Preoperative cervical MRI images, **e**–**f** preoperative cervical CT images, and **g**, **h** Postoperative cervical X-ray images taken one week after the procedure

Internal Fixation Device: The traditional titanium plate group consists of the Katia Anterior Cervical Internal Fixation System and iliac bone blocks for fusion. The door-shaped titanium plate group is composed of the door-shaped titanium plate (Figs. 1 and 2) and iliac bone blocks for fusion. The door-shaped titanium plate was developed and designed by Director Zhang Changjiang of our institution and has been granted national utility model patent ZL202120000201.7 and design patent ZL202130000119.X. Both types of plates were provided by Shanghai Sanyou Medical Devices Co., Ltd.

### Acquisition and selection of specimens

**Fig. 3:**
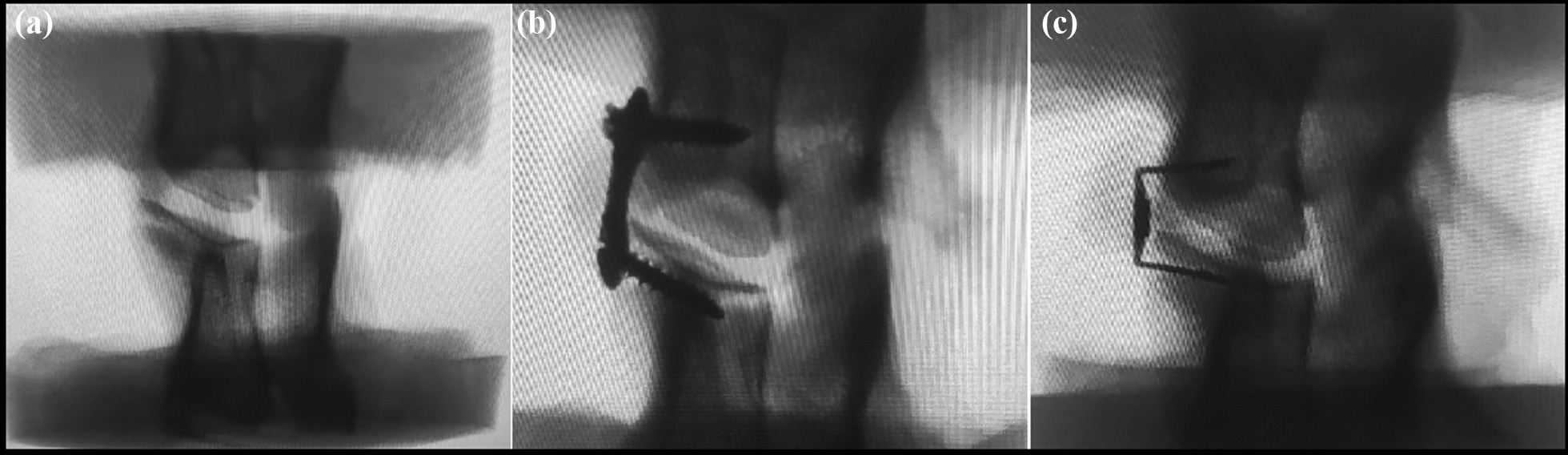
**a**, **b**, **c** Radiographs of specimens before internal fixation and after performing different internal fixations

The average weight of 10 mountain goats was 53.15 ± 3.83 kg, and specimens of the C4/5 vertebral segment were selected. All specimens underwent X-ray and bone density examinations to exclude abnormal specimens, such as tumours, bone destruction, trauma, degenerative changes, and osteoporosis. X-rays showed no obvious abnormalities in the specimens (Fig. 3), and bone density was within the normal range (1.651 ± 0.034 g/cm^3^). All specimens were carefully dissected to remove paravertebral muscles, anterior longitudinal ligaments, and fat tissues, while retaining appendages and major ligaments (anterior longitudinal ligament, posterior longitudinal ligament, ligamentum flavum, interspinous ligament, supraspinous ligament), joint capsules, and facet joints. Double-layer dressings were used to wrap and store the specimens at − 20 °C for later use in specimen model preparation.

### Model preparation

The upper 1/3 of the C4 vertebra and the lower 1/3 of the C5 vertebra were embedded in a mould using denture base resin, with the C4/5 vertebrae positioned at the centre of the mould, and the C4/5 intervertebral space parallel to the upper and lower modules formed by the denture base resin. After biomechanical experiments for all specimens before fusion fixation, 5 specimens were randomly selected for intervertebral disc removal, assisted by anterior titanium plate fixation and bone fusion, and designated as the research group. The remaining 5 specimens underwent intervertebral disc removal, assisted by the Katia cervical anterior fixation system and bone fusion, and were designated as the control group. Both groups of patients underwent X-ray examinations (Fig. 3), and X-rays showed that the implanted bone blocks were positioned in the intervertebral space, without entering the spinal canal, and the screws did not penetrate the endplate into the intervertebral space.

### Mechanical experiment and loading sequence

**Fig. 4:**
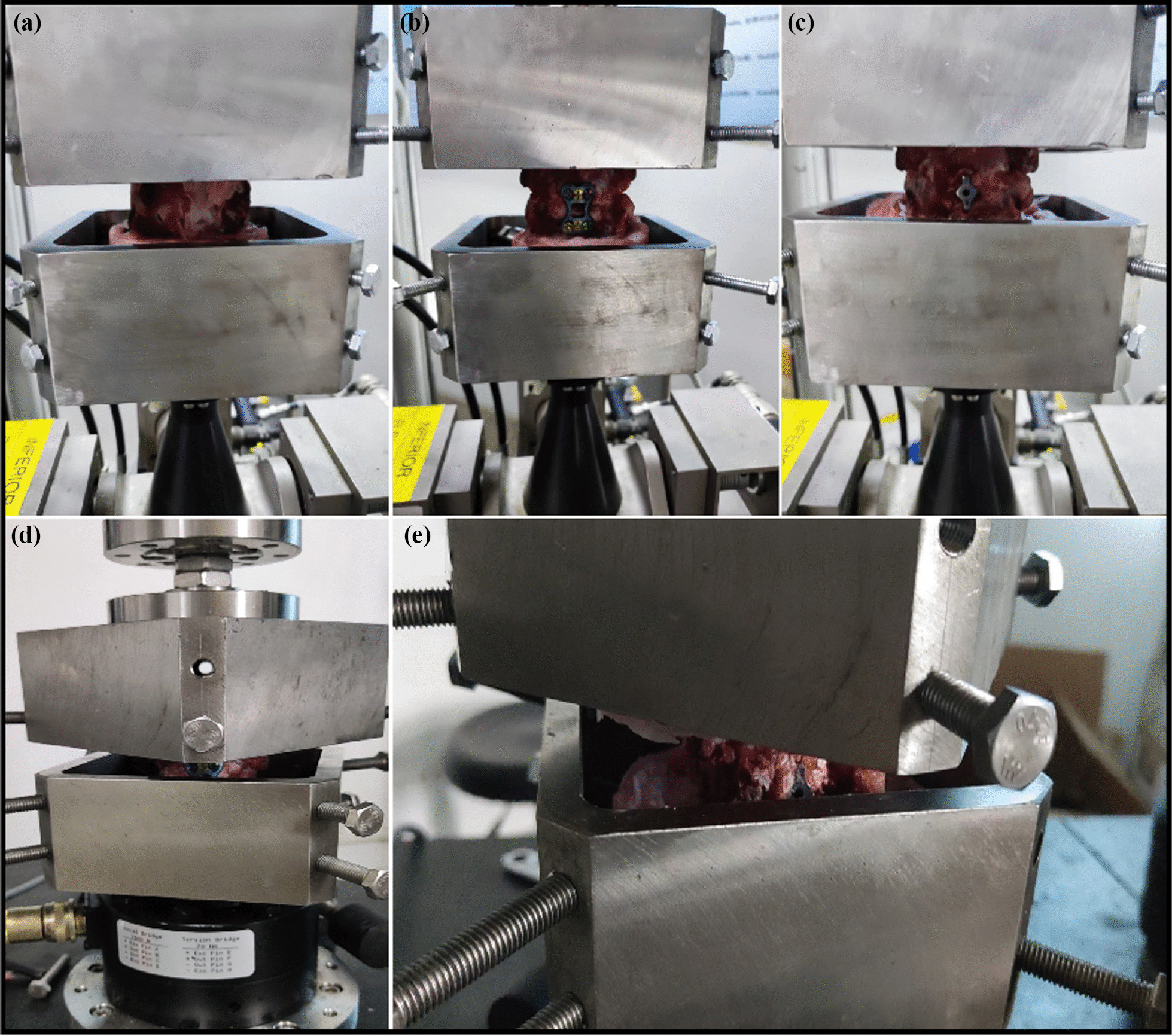
**a**, **b**, **c** Three-Dimensional Motion Stability Test was performed on specimens from the pre-fixation, post-fixation, and post-torsion groups; **d**, **e** After completion of post-fixation biomechanical testing 3000 torsions were performed on both groups of specimens

MTS was used to simulate the physiological motion of the cervical spine in six directions: flexion–extension, lateral bending (left and right), and axial rotation for 10 specimens. A maximum torque of 2.5 N·m and a vertical physiological load of 20 N were applied. To minimize experimental errors caused by creep, a preloading of three cycles was applied to the specimens before data acquisition. The specimens were then allowed to rest for 60 s until they returned to their neutral position before formal testing. MTS collected the spatial coordinates of the end effector of the mechanical arm and obtained the torque values at various data collection points on the data acquisition board. Comprehensive curve graphs were generated through computer processing, providing the range of motion (ROM) and neutral zone (NZ) in each direction. ROM and NZ values were obtained for all samples before fixation using the above-mentioned method. The two groups of samples were then fixed, and ROM and NZ were measured for the research group and the control group. Subsequently, both groups of samples were subjected to a torsional load with a frequency of 0.5 Hz, 3000 cycles, and a torque of 2 N m. ROM and NZ of the specimens were measured after torsion (Fig. 4). Throughout the experiment, intermittent spraying of physiological saline was applied to keep the specimens hydrated.

### Statistical analyses

All measurement results were statistically analysed using SPSS 26.0 software. Independent samples t tests were employed to compare ROM and NZ between the two groups after fixation, followed by paired t tests to compare the post-fixation data with the pre-fixation data. After 3000 cycles of torsion, independent samples t tests were performed again to compare ROM and NZ between the two groups, followed by paired t tests to compare the post-fixation and pre-fixation data, respectively. A significance level of *P* < 0.05 indicated statistical significance.

## Results

**Fig. 5:**
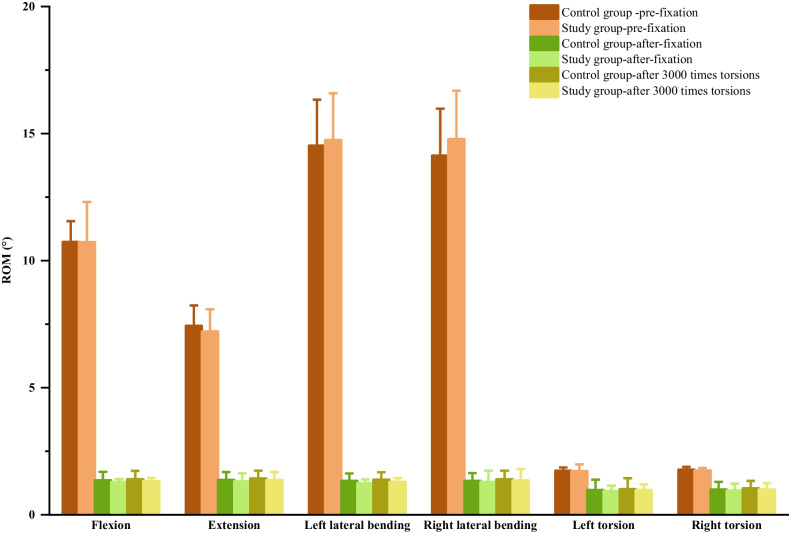
ROM graphs of two sample groups before and after internal fixation, as well as after torsion

**Fig. 6:**
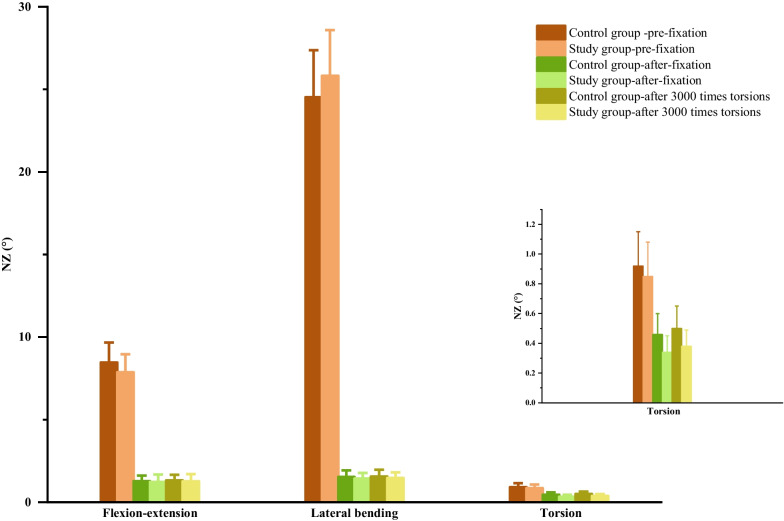
NZ graphs of two sample groups before and after internal fixation, as well as after torsion

All specimens exhibited normal morphology without bone damage, and intervertebral height was well preserved. Internal fixation in both groups effectively restored intervertebral height, reestablished the physiological curvature, and ensured proper bone placement, with secure fixation of adjacent vertebrae. There was no penetration of fixation screws into the spinal canal or endplates. Comparisons of ROM and NZ in six directions before fixation, after fixation, and after 3000 torsional cycles can be found in Tables 1 and 2, Figs. 5 and 6. The *p* values for all statistical analyses are provided in Tables 3 and 4. In both internal fixation groups, ROM and NZ in all directions significantly decreased compared to pre-fixation values, with statistical significance (*P* < 0.05). However, the research group exhibited slightly lower ROM and NZ in all directions compared to the control group, but the differences were not statistically significant (*P* > 0.05). After 3000 torsional cycles, ROM and NZ in all directions for both groups remained significantly lower compared to pre-fixation values (*P* < 0.05), with a slight increase compared to pre-torsion values (*P* < 0.05), but the magnitude of increase was minimal. In comparison, the research group still demonstrated slightly lower ROM and NZ in all directions compared to the control group, without statistical significance (*P* > 0.05).

**Table 1:** The ROM of C4/5 in different directions of two groups

Direction of movement	Control group			Study group		
	Pre-fixation	Post-fixation	After torsion	Pre-fixation	Post-fixation	After torsion
Flexion	10.73 ± 0.82	1.35 ± 0.33	1.40 ± 0.33	10.73 ± 1.58	1.28 ± 0.12	1.32 ± 0.13
Extension	7.43 ± 0.80	1.37 ± 0.30	1.43 ± 0.31	7.21 ± 0.88	1.31 ± 0.32	1.36 ± 0.31
Left lateral bending	14.52 ± 1.80	1.33 ± 0.29	1.38 ± 0.29	14.74 ± 1.84	1.24 ± 0.15	1.29 ± 0.15
Right lateral bending	14.13 ± 1.85	1.33 ± 0.31	1.39 ± 0.34	14.78 ± 1.91	1.29 ± 0.44	1.35 ± 0.44
Left axial rotation	1.73 ± 0.13	0.97 ± 0.41	1.01 ± 0.42	1.71 ± 0.27	0.93 ± 0.21	0.97 ± 0.22
Right axial rotation	1.77 ± 0.11	0.99 ± 0.3	1.04 ± 0.29	1.74 ± 0.10	0.95 ± 0.27	0.99 ± 0.25

**Table 2:** The NZ of C4/5 in different directions of two groups

Direction of movement	Control group			Study group		
	Pre-fixation	After fixation	After torsion	Pre-fixation	After fixation	After torsion
Flexion–Extension	8.47 ± 1.2	1.28 ± 0.34	1.32 ± 0.34	7.87 ± 1.09	1.23 ± 0.44	1.26 ± 0.44
Left–Right lateral bending	24.53 ± 2.84	1.53 ± 0.4	1.56 ± 0.41	25.82 ± 2.77	1.43 ± 0.34	1.47 ± 0.34
Left–Right axial rotation	0.92 ± 0.23	0.46 ± 0.14	0.50 ± 0.15	0.85 ± 0.23	0.34 ± 0.11	0.38 ± 0.11

**Table 3:** *P* values after statistical analysis of ROM

	Flexion	Extension	Left lateral bending	Right lateral bending	Left axial rotation	Right axial rotation
Control group versus Study group (pre-fixation)	0.994	0.682	0.855	0.599	0.862	0.641
Control group versus Study group (after fixation)	0.671	0.79	0.542	0.884	0.866	0.812
Control group versus Study group (after torsion)	0.642	0.757	0.565	0.888	0.876	0.799
Pre-fixation versus after fixation (Control group)	< 0.001	< 0.001	< 0.001	< 0.001	0.014	0.002
After fixation versus after torsion (Control group)	0.001	0.003	0.011	0.009	0.008	0.007
Pre-fixation versus after torsion (Control group)	< 0.001	< 0.001	< 0.001	< 0.001	0.019	0.003
Pre-fixation versus after fixation (Study group)	< 0.001	< 0.001	< 0.001	< 0.001	0.012	0.004
After fixation versus after torsion (Study group)	0.019	0.005	0.006	0.008	0.004	0.002
Pre-fixation versus after torsion (Study group)	< 0.001	< 0.001	< 0.001	< 0.001	0.019	0.006

**Table 4:** *P* values after statistical analysis of NZ

Control group versus Study group (pre-fixation)	Flexion–extension	Left–right lateral bending	Left–right axial rotation
Control group versus Study group (pre-fixation)	0.434	0.639	0.65
Control group versus Study group (after fixation)	0.846	0.698	0.179
Control group versus Study group (after torsion)	0.822	0.71	0.175
Pre-fixation versus after fixation (Control group)	< 0.001	< 0.001	0.002
After fixation versus after torsion (Control group)	0.003	0.007	0.001
Pre-fixation versus after torsion (Control group)	< 0.001	< 0.001	0.003
Pre-fixation versus after fixation (Study group)	< 0.001	< 0.001	0.005
After fixation versus after torsion (Study group)	0.003	0.001	0.005
Pre-fixation versus after torsion (Study group)	< 0.001	< 0.001	0.008

## Discussion

Anterior cervical discectomy and fusion (ACDF) is a widely accepted and safe surgical approach for treating degenerative cervical spine diseases. Initially, stand-alone cages were used, which not only restored intervertebral space height but also promoted bone fusion [19]. However, challenges such as cage migration, subsidence, and segmental kyphosis have been encountered [20, 21]. In order to achieve better clinical outcomes, various anterior cervical fixation plates have been developed, including the AO cervical spine locking plate, zero-profile structure, dynamic plate, and cervical disc replacement. Stephen A. Smith and colleagues conducted non-destructive testing on the cervical specimens of 5 fresh cadavers implanted with the AO cervical spine locking plate. The results indicated significant stability during flexion but insufficient stability during rotation [22]. A 4-year follow-up study by Barbagallo et al. reported that the zero-profile structure is safe and effective for both single-level and multi-level ACDF [23]. Chang BQ, through clinical research, found that the incidence of swallowing difficulties in patients using the Zero-profile device and traditional nail plate system was 9.52% and 29.17%, respectively, with a significant difference between the two groups [24]. However, Lee et al., through tracking and analysing clinical data of patients who underwent single-level interbody fusion, found that the zero-profile structure was inferior to the cage and plate structure in maintaining spinal structure and stability, preventing subsidence, and achieving bone fusion [12]. Pitzen et al. [25], through a clinical study of 132 patients, found that the risk of complications such as plate fracture and screw loosening with dynamic plate was lower than with traditional plates, and bone fusion occurred faster. However, there are also reports indicating that patients using dynamic plates have a higher rate of graft subsidence, accompanied by the loss of cervical physiological curvature [26]. Cervical disc replacement differs significantly from the anterior cervical plate system; it almost does not restrict cervical spine movement, avoiding the risk of postoperative loss of cervical range of motion and also reducing the probability of swallowing difficulties. However, it is also associated with potential risks such as accelerated degeneration of the facet joints, nonphysiological motion, and particulate wear [27]. In summary, although these plates have unique advantages, their shortcomings are also evident, such as the complexity of operations with too many screws, significant loss of normal cervical physiological curvature, and a decrease in intervertebral space height.

In addition, the choice of screws is crucial for the stability of the surgically fixed site. Unicortical screws and bicortical screws are frequently used in orthopaedic surgery, and many scholars have compared and analysed their fixation effects. Pater TJ et al. randomly allocated six pairs of matched fresh-frozen cadaver forearms into a unicortical locking group and a bicortical unlocking group, conducting bending and twisting tests to obtain structural stiffness and fixation strength. The results showed equivalent fixation strength in the two groups under bending, with the unicortical locking group having higher bending stiffness. However, the stiffness and strength of this group were significantly lower during twisting [28]. Little et al., through biomechanical studies on a model of externally fixed clavicular fractures in 15 cases, aimed to determine the effectiveness of three internal fixation methods: unicortical + locking plate, bicortical + locking plate, and bicortical + non-locking plate. The conclusion drawn was that the quantity of cortical bone has the greatest impact on fixation effectiveness. Unicortical locking plates may fail at significantly lower loads and stress levels. Bicortical fixation is significantly better than unicortical fixation, and whether the fixation device is a locking device does not significantly affect fixation effectiveness [29]. Sun et al. conducted a retrospective analysis of 12 cases of occipitocervical revision surgery using a bicortical screw and plate system from 2010 to 2018. These surgeries employed depth-limited drilling, progressively deepening the channel to reach the internal bone plate. Simultaneously, a probe was used to explore all walls of the channel, and finally, bicortical screws were inserted into the occipital bone plate. All patients achieved good fixation and bone fusion postoperatively, without major complications. Clinical symptoms were significantly relieved, leading to the conclusion that the posterior occipitocervical screw-plate system using bicortical screws has advantages such as safety, simplicity, and good efficacy, making it valuable for improving occipitocervical revision surgery [30]. These studies collectively indicate that bicortical screws have better fixation effects. In ACDF surgery, due to the thicker vertebral bodies and the presence of the spinal cord within the vertebral canal, it may be challenging to control the length of screws when using bicortical screws. Therefore, the current use of unicortical screws in the door-shaped titanium plate is understandable. However, in future development, the application of depth-limited drilling and probe can be employed to gradually deepen the channel and promptly investigate the internal conditions of the channel. Subsequently, the screws in the door-shaped titanium plate can be modified to bicortical screws, thereby further enhancing fixation stability under safe conditions.

In this experiment, a non-destructive approach was employed to evaluate the immediate stability performance of the internal fixation model. This design offers several advantages: (1) the application of axial load can compress the specimen, better simulating the loading conditions of the cervical spine in the human body. (2) Precise adjustment of loading in various directions was achieved through MTS, avoiding load loss caused by coupled movements and enabling synchronized data acquisition with torque loading, reducing experimental errors. (3) In contrast to most cervical spine mechanical experiments using 1.5 N·m as the motion load, this experiment, aimed at further verifying the mechanical performance of the door-shaped titanium plate, used 20 N as the axial load and 2.5 N·m as the motion load. (4) In addition to using the classic range of motion (ROM) as an indicator for stability assessment, this study also employed neutral zone (NZ). NZ represents the region where the spinal motion segment moves essentially free of applied loading, requiring minimal postural muscular effort [31–33]. In quantifying stability, it is often superior to ROM because it has higher sensitivity and can be applied to any setting of maximum loading bounds, even if the load–deflection curve is asymmetrical, it does not affect its use [33, 34]. (5) This experiment introduced short-frequency activities within the physiological load range, which could detect the immediate stability performance of both groups of cervical spines after performing a certain frequency of torsion, making the experimental results more convincing.

The results of this experiment show that, compared to pre-fixation, the research group post-fixation exhibited a significant decrease in ROM and NZ in all six directions, and they followed a consistent trend: flexion and extension ROM decreased by 88.07% and 81.83% respectively, while NZ decreased by 84.37%. Lateral bending ROM decreased by 91.59% on the left and 91.27% on the right, with NZ decreasing by 94.46%. Torsional ROM decreased by 45.61% on the left and 45.40% on the right, with NZ decreasing by 60.00%. In contrast, the control group, relative to pre-fixation, showed a decrease of 87.42% in flexion and 81.56% in extension ROM, with NZ decreasing by 84.89%. Lateral bending ROM decreased by 90.84% on the left and 90.59% on the right, with NZ decreasing by 93.76%. Torsional ROM decreased by 43.93% on the left and 44.07% on the right, with NZ decreasing by 50.00%. We can observe that both groups were able to effectively restore the stability of unstable cervical spine specimens, and the performance of the door-shaped titanium plate was slightly superior to traditional titanium plates. Crawford et al.’s [35] biomechanical experimental results suggest that two fixation screws combined with a titanium alloy plate can achieve similar biomechanical performance as a four-screw combined titanium plate, which is consistent with the results obtained in this experiment.

Short-frequency activities within the physiological load range can also serve as one of the methods for evaluating the immediate stability performance of cervical ACDF surgery [36]. Until the implanted bone blocks fully fuse, the stability of the operative segment of the cervical spine still relies on the internal fixation device to maintain it. Postoperatively, patients cannot avoid some degree of torsional movement, even with the use of a neck brace. In this experiment, after subjecting the internal fixation group to a torsional load of 2 N m, 0.5 Hz, and 2000 cycles, we again measured and recorded the ROM and NZ of each group. We found that the mechanical parameters of both groups had increased slightly, but still significantly decreased compared to the physiological group, with statistical significance. Furthermore, there were no statistically significant differences between the groups. There were no signs of internal fixation device failure during the experimental loading process. This indicates that both groups possess reliable immediate stability performance, and a certain frequency of torsional activity does not affect their stability performance.

Compared to the traditional titanium plate, the door-shaped titanium plate significantly reduces the incidence of complications. Complications associated with the traditional titanium plate primarily focus on difficulties in swallowing, loosening or fracture of screws, adjacent segment diseases, adjacent segment ossification, and occurrence rates [37–39]. According to previous literature, difficulty in swallowing is the most common and direct postoperative complication, with an incidence ranging from 1.7 to 67% [40, 41]. Factors such as the number of involved segments, surgical duration, plate size, and the extent of tissue dissection and retraction are considered potential contributors to swallowing difficulties, but the primary factor is the thickness of the plate [42, 43]. Lee and others, by comparing titanium plates of different thicknesses, believed that the thickness and texture of the titanium plate are directly related to swallowing discomfort. Thinner and smoother titanium plates exert less irritation to soft tissues and the oesophagus, resulting in a lower incidence of postoperative swallowing discomfort [44]. The door-shaped titanium plate, with a thickness of only 2 mm and a smooth, flat surface, reduces postoperative swallowing discomfort in patients. The door-shaped titanium plate’s plate and screws are made from a single piece of titanium alloy without gaps at the joints, thereby preventing screw loosening. The extremely high strength and hardness of titanium alloy make screw fracture unlikely. This theoretically allows the door-shaped titanium plate to ensure cervical stability for a longer duration, although this requires further destructive testing to confirm. Furthermore, since it does not require frequent screw installation, there is an increasing amount of clinical and radiological evidence indicating the accelerated degeneration of adjacent fusion segments of the spinal column [45, 46]. Changes in adjacent segment degeneration (ASD) may be related to the biomechanical effects of cervical fusion and/or the biological effects of cervical degeneration [47–49]. After fusion, changes in the biomechanics of adjacent segments can lead to increased mobility [50], increased load [51], or increased intervertebral disc pressure [52], ultimately accelerating disc degeneration [53, 54]. Clinical risk factor analysis of ASD following ACDF suggests that the plate-to-disc distance (PDD) may be an independent triggering factor for ASD [46, 55–57]. Chung and colleagues conducted follow-ups of over 10 years on 177 ACDF patients and found that most clinical cases of ASD occurred in patients with a PDD less than 5 mm. Therefore, they believe that a PDD greater than or equal to 5 mm is favourable for preventing ASD [46]. In addition, Yu et al. performed logistic regression analysis on 138 patients and also concluded that PDD less than 5 mm is a risk factor for ASD [58]. Similarly, adjacent segment ossification development (ALOD), another common complication of ACDF, can occur as early as 3 months postoperatively, and it is also associated with PDD [59]. Park and colleagues investigated the incidence of ALOD after ACDF, and their findings indicated that ALOD is more likely to occur when the distance between the ends of the plate and adjacent intervertebral discs is less than 5 mm [57, 60]. This suggests that controlling PDD to be greater than or equal to 5 mm is of significant importance in reducing the incidence of both ASD and ALOD. Compared to the larger and longer traditional titanium plates, the door-shaped titanium plate has a length of only 14–18 mm. After adapting to different sizes of cervical vertebrae, it meets the requirement of a PDD greater than 5 mm between the ends of the plate and adjacent intervertebral discs, significantly reducing the incidence of ASD and ALOD.

With the development of minimally invasive techniques for the posterior approach to the cervical spine, there is growing interest in endoscopic techniques for the anterior cervical spine. The primary focus is on percutaneous endoscopic anterior cervical discectomy (PEACD) [61, 62]. This procedure involves the endoscopic removal of cervical intervertebral discs from the anterior route, achieving complete spinal nerve decompression by percutaneously removing the responsible disc segments. However, because PEACD does not involve intervertebral fusion during the procedure, it has certain limitations. These include the inability to provide stability for the cervical surgical segment, the inability to restore the physiological curvature of the cervical spine, a higher risk of recurrence compared to fusion surgery, and a certain degree of height loss in the intervertebral space of the responsible segment. Therefore, it has a limited range of indications and is only suitable for soft intervertebral disc protrusions resulting from cervical injuries, CSM, CSR, and similar conditions. Patients with cervical diseases such as intervertebral disc displacement, calcified disc protrusions, ossification of the posterior longitudinal ligament, and narrow intervertebral disc spaces (less than 3 mm), as well as patients with neurological or vascular disorders similar to intervertebral disc protrusions, are not suitable candidates for this procedure [63, 64]. In comparison to PEACD, the development of mini open (air/water medium) endoscopy-assisted anterior cervical discectomy and fusion (MOEA-ACDF) has been relatively slow. This is because traditional titanium plates are large and not suitable for clamping, and they require frequent screwing, making them unsuitable for MOEA-ACDF. The door-shaped titanium plate is thinner and smaller, making it convenient for endoscopic clamping. It combines the plate and screws, reducing the intraoperative screw locking process, and can be easily hammered in with a bone hammer in just 10 s. Additionally, the screw foot has a spiky shape, allowing it to securely fasten to the vertebrae and apply continuous pressure to the vertebrae, promoting bone fusion. Therefore, it can fully function in MOEA-ACDF. MOEA-ACDF, based on intervertebral disc removal, enhances cervical stability through fusion, improving surgical effectiveness. Compared to PEACD, it broadens the range of indications for minimally invasive techniques, making them applicable to a wider range of cervical conditions.

## Limitations

This study has experimentally demonstrated that the door-shaped titanium plate has many advantages. However, there are still the following shortcomings that need to be considered and addressed: (1) This experiment was only conducted on goat cadaver specimens and did not involve human cadaver experiments, making it unable to fully simulate the actual spinal motion in the human body, requiring further experimental research; (2) this experiment may have situations that were not observed due to the limited sample size; and (3) destructive experiments were not conducted, and the experimental results could not verify the maximum resistance to device failure.

## Conclusions

In summary, this study confirms that the door-shaped titanium plate has mechanical properties similar to traditional titanium plates in all directions. Moreover, its smaller size and simpler surgical procedure make it suitable for cervical anterior endoscopic surgery, reducing surgical trauma and demonstrating clinical feasibility, worthy of further research and promotion.

## Data Availability

The datasets used and/or analysed during the current study are available from the corresponding author on reasonable request.
